# ER stress-driven unfolded protein response fuels aging-related tumor aggressiveness in gliomas

**DOI:** 10.3389/fmolb.2025.1640038

**Published:** 2025-07-11

**Authors:** Xiaodong Shao, Shaolei Guo, Jia Yang, Junjie Dai, Kaihua Cao, Xia Cai, Tianshi Song, Shun Yao, Umar Raza, Kun Chen

**Affiliations:** ^1^ Department of Neurosurgery, The First Affiliated Hospital, Sun Yat-Sen University, Guangzhou, Guangdong, China; ^2^ Department of Neurosurgery, Ninth People Hospital Affiliated to Shanghai Jiao Tong University School of Medicine, Shanghai, China; ^3^ Rongchang Biopharmaceutical (Yantai) Co., Ltd., Yantai, Shandong, China; ^4^ Department of Neurology, The First Affiliated Hospital, Harbin Medical University, Harbin, Heilongjiang, China; ^5^ Department of Thoracic Surgery, Affiliated Hospital of Guangdong Medical University, Zhanjiang, Guangdong, China

**Keywords:** glioma, aging, tumor aggressiveness, ER stress, UPR

## Abstract

**Background:**

Gliomas are the most prevalent and aggressive primary brain tumors. Aging significantly influences glioma incidence and progression, yet the molecular mechanisms linking aging-related pathways to tumor aggressiveness remain poorly understood. Here, we aimed to decipher aging-related molecular mechanisms regulating tumor aggressiveness in gliomas.

**Methods:**

We performed comprehensive aging-targeted transcriptomic analyses using TCGA-glioma patient dataset. Differential gene and protein expression, functional annotation and pathway enrichment, gene set enrichment, network construction, CRSISPR-based functional dependency, transcription factor prediction, correlation, clinical association and survival analyses were conducted to identify, develop and validate endoplasmic reticulum (ER) stress-driven unfolded protein response (UPR) as key aging-related molecular mechanism driving tumor aggressiveness in gliomas. Notably, we validated our findings in multiple independent GEO datasets.

**Results:**

We identified ER stress and UPR as key aging-related mechanism behind tumor aggressiveness in gliomas, and developed a six gene “ER Stress and UPR-driven Aging-related Tumor Aggressiveness in Glioma” (ESURATAG) gene signature, comprising DERL2, RPN2, SEC13, SEC61A1, SEC61B, and STT3A. Notably, glioma cell proliferation critically depends on ESURATAG-GS, which is preferentially regulated by MYC and is associated with disease and cell cycle progression, inflammation, and poor clinical outcomes in glioma patients, simultaneously aligning with aging and tumor aggressiveness signatures. Validated in multiple GEO datasets, high ESURATAG expression is linked to disease onset, advanced disease state, and reduced overall and progression-free survival in glioma patients as well as in patients with major subtypes of gliomas, including oligodendrogliomas, astrocytomas and gliobalstomas.

**Discussion:**

ESURATAG-GS serves as a critical MYC-regulated adaptive mechanism that fuels aging-related tumor aggressiveness via ER stress-driven UPR in gliomas, presenting novel prognostic markers and therapeutic targets for elderly glioma patients.

## 1 Background

Cancer poses a major global health challenge and remains one of the leading causes of mortality worldwide (Bray et al., 2024). Gliomas are the most prevalent and aggressive primary brain tumors, accounting for approximately 30% of all brain tumors and over 80% of malignant brain neoplasms worldwide (Wang et al., 2022). These tumors encompass a heterogeneous group, ranging from relatively indolent low-grade gliomas to the highly malignant glioblastomas (GBMs) characterized by rapid progression, therapeutic resistance, and dismal patient survival (Forst et al., 2014). Moreover, gliomas exhibit complex interactions with the tumor microenvironment, which includes non-neoplastic cells, extracellular matrix components, and diverse molecular stress signals, all of which influence tumor growth and invasion (Jang and Park, 2025; Zhang et al., 2025b). Despite considerable advances in neurosurgical techniques, radiotherapy, and chemotherapy, glioma patient outcomes have remained largely stagnant (van Solinge and Nieland, 2022), highlighting the urgent need for a deeper molecular understanding of tumor progression and aggressiveness in gliomas to guide novel therapeutic approaches and improve patient outcomes.

Aging is a major risk factor for cancer development, as it is associated with the accumulation of genetic mutations, chronic inflammation, and declines in immune surveillance that collectively create a permissive environment for tumor initiation and progression (Leonardi et al., 2018; López-Otín et al., 2023b). Likewise, aging is a significant risk factor for glioma incidence and progression, profoundly influencing tumor biology and patient prognosis (Jia et al., 2022). Several studies have documented a higher incidence of gliomas, particularly GBMs, in the elderly population. For instance, over half of newly diagnosed GBM patients are over the age of 65, with a peak incidence between ages 75 and 84 (Chahal et al., 2022). This increasing incidence in the elderly population highlights the growing clinical challenge posed by gliomas in aging societies. Likewise, older patients with GBM experience severe disease and poor clinical outcomes, as patients aged ≥65 years have a median OS of about 7.2 months, notably lower than that of younger cohorts (Cao et al., 2023). At molecular level, the aging brain undergoes multifaceted molecular and cellular changes, such as genomic instability, telomere attrition, epigenetic drift, and chronic low-grade inflammation, which contribute to altered tissue homeostasis and increased susceptibility to malignancies (López-Otín et al., 2023a; Jiang et al., 2025). Aging influences tumor cell-intrinsic pathways governing metabolism, proteostasis, and stress responses, which are critical for tumor adaptation and survival under hostile microenvironmental conditions (Shen et al., 2022; Sedrak and Cohen, 2023). However, the molecular underpinnings linking aging to tumor aggressiveness in gliomas remain poorly characterized, posing a barrier to developing aging-tailored treatment strategies.

Here, we aimed to identify aging-related molecular mechanisms regulating tumor aggressiveness in gliomas, and performed a comprehensive aging-targeted transcriptomic analysis to identify underlying molecular mechanisms and hubs. In brief, our results establish endoplasmic reticulum stress-driven unfolded protein response (UPR) as a key oncogenic pathway fueling aging-related tumor aggressiveness in gliomas and suggest that targeting this axis may offer novel therapeutic opportunities.

## 2 Methods

### 2.1 Data retrieved

Gene expression, gene methylation, and protein abundance data for glioma patients were obtained from The Cancer Genome Atlas (TCGA) via the Broad GDAC Firehose platform (https://gdac.broadinstitute.org/). Mutation profiles were accessed through the cBioPortal for Cancer Genomics (https://www.cbioportal.org/). Additional glioma expression datasets were collected from the Gene Expression Omnibus (GEO) including GSE4290 (Sun et al., 2006), GSE7696 (Nguyen et al., 2023), GSE10878 (de Tayrac et al., 2009), GSE16011 (Gravendeel et al., 2009), GSE21354 (Liu et al., 2011), GSE43289 (Vital et al., 2010), GSE43378 (Kawaguchi et al., 2013), GSE45921 (Zhou et al., 2013), GSE52009, GSE54004, GSE83300 (Feng et al., 2017), and GSE107850 (Gao et al., 2018). Protein expression data were sourced from the Clinical Proteomic Tumor Analysis Consortium (CPTAC) database using cProcite (https://cprosite.ccr.cancer.gov/). Functional dependency scores derived from CRISPR-based Chronos screens for genes within the UPR gene signature were retrieved for glioma cell lines from the DepMap portal (https://depmap.org/portal/), where negative scores indicate gene essentiality and scores near zero denote non-essential genes. Information regarding MYC transcription factor binding sites within the ESURATAG gene set was obtained from ChIPBase v3.0 (https://rnasysu.com/chipbase3/).

### 2.2 Patients’ classification

The list of human aging-related genes was obtained from the Human Aging Genomic Resource Database (https://genomics.senescence.info/gene_expression/signatures.html). For each patient, we calculated the Z-scores of all 307 genes included in this signature and then summed these values to derive an overall aging score. Patients were subsequently ranked based on their aging scores and categorized into three equally sized groups, low, intermediate, and high, for differential gene expression analyses. Additionally, for gene set enrichment analysis (GSEA) within primary tumors, patients were divided into two groups based on low and high aging scores. Separately, patients were also stratified into three age brackets: younger than 40 years, between 40 and 65 years, and older than 65 years, to further assess differential expression. Due to incomplete availability of clinical data on tumor pathological stage and metastatic status in the TCGA glioma dataset, we instead employed epithelial-mesenchymal transition (EMT) as a marker of tumor aggressiveness and metastatic potential, as supported by prior studies (Yang and Weinberg, 2008; Voulgari and Pintzas, 2009; Sethi et al., 2011; Andergassen et al., 2018). That’s why, tumor tissues were planned to be classified into low, intermediate and high EMT groups using gene signature based classification. We downloaded Hallmark_EMT gene signature from GSEA portal at https://www.gsea-msigdb.org/ and calculated Z-scores of all the 200 genes in this signature for each patient. Later, Z-scores were summed to calculate the EMT score for each patient. Lastly, patients were sorted according to their EMT score and were divided into three equal groups; low, intermediate and high, for differential expression analysis, and into two groups; low and high, for GSEA within primary tumors.

### 2.3 Identification of differentially expressed aging-related tumor aggressiveness hits

We analyzed mRNA expression levels of all genes across comparisons (1) between normal tissues and primary tumors, (2) among groups stratified by aging score, (3) among patient age categories, (4) among groups defined by EMT scores, and (5) between primary and recurrent tumors. A false discovery rate (FDR) threshold of less than 0.05 was applied to identify statistically significant differences. Genes that were consistently downregulated in primary tumors relative to normal tissue, showed progressive downregulation from low to high aging score groups, from younger to older patients, from low to high EMT score groups, and were further downregulated in recurrent tumors compared to primary tumors were classified as aging-related tumor suppressor genes associated with tumor aggressiveness in gliomas. Conversely, genes exhibiting the opposite pattern, being upregulated in primary tumors versus normal tissue, progressively increased expression from low to high aging score groups, from young to old, from low to high EMT score groups, and further upregulated in recurrent tumors compared to primary tumors—were designated as aging-related oncogenic hits associated with glioma aggressiveness.

### 2.4 Functional annotation, pathway enrichment, network construction and gene signature development

Functional annotation and pathway enrichment analyses were conducted using the freely accessible DAVID functional annotation tool (https://davidbioinformatics.nih.gov/). The sets of aging-related tumor suppressor and oncogenic genes associated with tumor aggressiveness were separately submitted to DAVID to determine their involvement in KEGG pathways, biological processes (BP), cellular components (CC), and molecular functions (MF). A significance threshold of p-value<0.05 was applied. Genes enriched in ER stress and UPR-related pathways were then selected and uploaded to the STRING database (https://string-db.org/) to construct a protein-protein interaction network. From this network, the interacting gene cluster was consolidated to define the ER Stress and Unfolded protein Response-driven Aging-related Tumor Aggressiveness in Glioma (ESURATAG) gene signature.

### 2.5 Gene set score calculation and GSEA

Gene set scores for individual patients were computed by summing the Z-scores of all genes within each specified gene set. GSEA was then conducted using curated gene sets associated with glioma, ER stress, UPR, MYC targets, and disease progression, obtained from the official GSEA database (http://software.broadinstitute.org/gsea/index.jsp). A significance threshold of p-value<0.05 was applied. The normalized enrichment scores (NES) alongside their corresponding p-values from each GSEA were visualized using GraphPad Prism v6.

### 2.6 Transcription factor analysis

To identify potential transcription factors (TFs) regulating the ESURATAG gene signature, the gene list was submitted to publicly accessible TF prediction platforms, Chea3 (https://maayanlab.cloud/chea3/) and hTFtarget (http://bioinfo.life.hust.edu.cn/hTFtarget#!/). The resulting TF predictions from both databases were then compared and integrated to determine shared transcriptional regulators of the input genes.

### 2.7 Survival analyses

Kaplan-Meier survival analyses were performed using custom R scripts, excluding patients lacking survival time or event data from the respective cohorts. Group stratification was determined based on the optimal cut-off threshold. Differences in survival between groups were assessed using the Log-rank (Mantel-Cox) test, with statistical significance set at p < 0.05. The resulting survival curves were subsequently reproduced and visualized in GraphPad Prism v6.

### 2.8 Statistical analyses

To compare differences between two groups, Student’s t-test was applied, while one-way ANOVA was used for comparisons involving more than two groups. Pearson correlation coefficients were calculated to assess relationships between variables. Visualization of bar graphs and dot plots was performed using GraphPad Prism v6. Venn diagrams were generated with an online tool available at http://bioinformatics.psb.ugent.be/webtools/Venn/.

## 3 Results

### 3.1 Aging-related tumor aggressiveness-targeted differential expression analyses identifies ER-stress and UPR as key oncogenic signaling pathways in glioma patients

**FIGURE 1:**
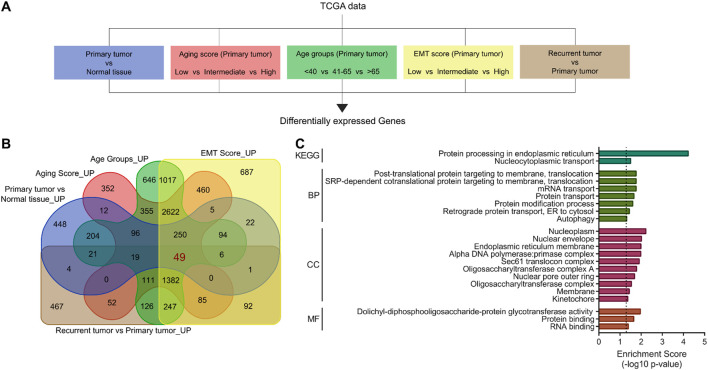
Aging-related tumor aggressiveness-targeted differential expression analyses identifies ER-stress and UPR as key oncogenic signaling pathways in glioma patients. **(A)** Illustration showing analysis pipeline used to identify molecular hubs associated with aging-related tumor aggressiveness in gliomas. Briefly, a set of differential gene expression analyses were performed in gliomas, comparing gene expression (1) between normal tissues and primary tumors, (2) among groups stratified by aging score, (3) among patient age categories, (4) among groups defined by EMT scores, and (5) between primary and recurrent tumors. **(B)** Venn diagram showing number of aging-related tumor aggressiveness-associated oncogenic hubs through pipeline in **(A)**. **(C)** Bar-graph showing enrichment score of KEGG pathways, biological processes (BP), cellular compartments (CC) and molecular functions (MF) in which oncogenic hubs, identified in **(B)**, are enriched. EMT, epithelial-to-mesenchymal transition.

In order to identify the Aging-related tumor aggressiveness associated pathways in gliomas, we first downloaded the gene expression data of gliomas from TCGA dataset, and performed differential expression analyses (1) between normal and primary tumor tissues, (2) among low, intermediate and high aging score groups (For details: see Materials and Methods section), and (3) among young-to-old age patients, (4) among low, intermediate and EMT score groups (For details: see Materials and Methods section), and (5) between primary and recurrent tumors (Figure 1A). As a result of these analyses, we found that 67 tumor suppressor hits being successively downregulated in above-mentioned differential expression analyses (Supplementary Figure S1A), and 49 oncogenic hits being successively upregulated in above-mentioned differential expression analyses (Figure 1B), were associated with aging-related tumor aggressiveness in gliomas. Next, we performed functional annotation and pathway enrichment analyses to identify the processes and pathways driven by these tumor suppressor and oncogenic hits. In this lines, we found that tumor suppressor hits were primarily enriched in pathways related normal brain functioning (Supplementary Figure S1B), whereas oncogenic hits were primarily enriched in pathways associated with ER stress and UPR (Figure 1C). Based on its well-established oncogenic and tumor promoting role, as well as general inclination towards pharmacological targeting oncogenic hubs rather than boosting tumor suppressor pathways, we aimed to focus on ER stress-related UPR for subsequent analyses.

### 3.2 ER stress and UPR-related gene signatures align with aging and tumor aggressiveness in glioma patients

**FIGURE 2:**
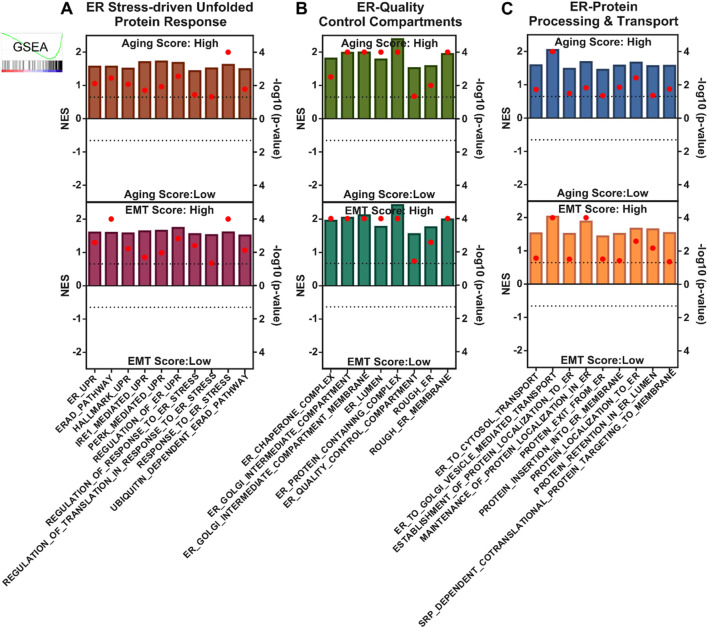
ER stress and UPR-related gene signatures align with aging and tumor aggressiveness in glioma patients. **(A–C)** Combined bar-dot-plots showing normalized enrichment scores (NES) (bars) and enrichment significance (dots) of GSEAs of ER stress-driven UPR-related **(A)**, ER-quality control compartments-related **(B)** and ER-based protein processing and transport-related **(C)** gene signatures in glioma patients having low vs. high aging scores (top) and low vs. high EMT scores (bottom) in TCGA dataset. EMT; epithelial-to-mesenchymal transition, ER; endoplasmic reticulum.

Next, we sought to confirm whether ER stress and UPR truly characterize aging-related tumor aggressiveness in glioma patients. To this end, we performed GSEA and found that different gene sets associated with ER-driven UPR were enriched in patients having high aging scores compared to those having low aging scores as well as in patients with high EMT scores compared to those with low EMT scores (Figure 2A). In addition, different gene sets associated with quality control compartments of ER were also enriched in patients having high aging scores compared to those having low aging scores, as well as in patients with high EMT scores compared to those with low EMT scores (Figure 2B). Furthermore, different gene sets associated with protein processing and transport by ER were also enriched in patients having high aging scores compared to those with low aging scores, as well as in patients having high EMT scores compared to those with low EMT scores (Figure 2C). Overall, these findings confirm that ER stress-driven UPR is key molecular mechanism behind aging-related tumor aggressiveness in glioma patients.

### 3.3 Glioma cells are preferentially dependent on ER stress and UPR-driven aging-related tumor aggressiveness in glioma (ESURATAG) gene signature for proliferation and survival

**FIGURE 3 F3:**
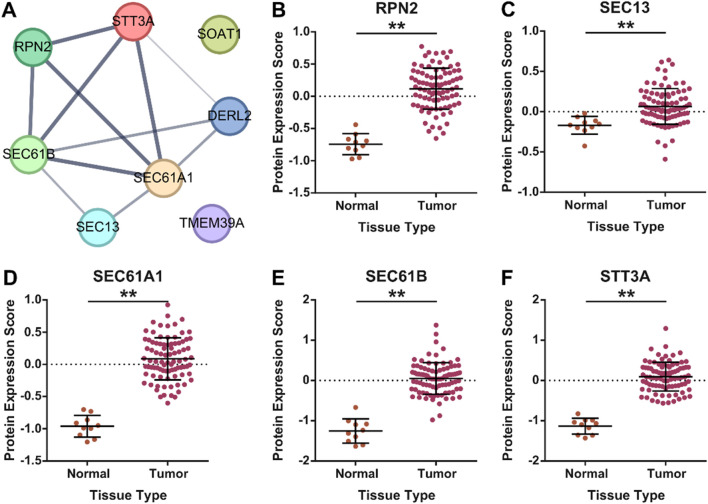
ESURATAG-GS development and protein expression of genes comprising ESURATAG-GS in gliomas. **(A)** Image showing String network of ER stress and UPR-related genes from functional annotation and pathway enrichment analyses. Linked nodes were combined to devise ESURATAG-GS. **(B–F)** Dot-plots showing changes in protein expression of RPN2 **(B)**, SEC13 **(C)**, SEC61A1 **(D)**, SEC61B **(E)**, and STT3A **(F)** between normal tissues and gliomas from CPTAC database. **: p < 0.01.

**FIGURE 4 F4:**
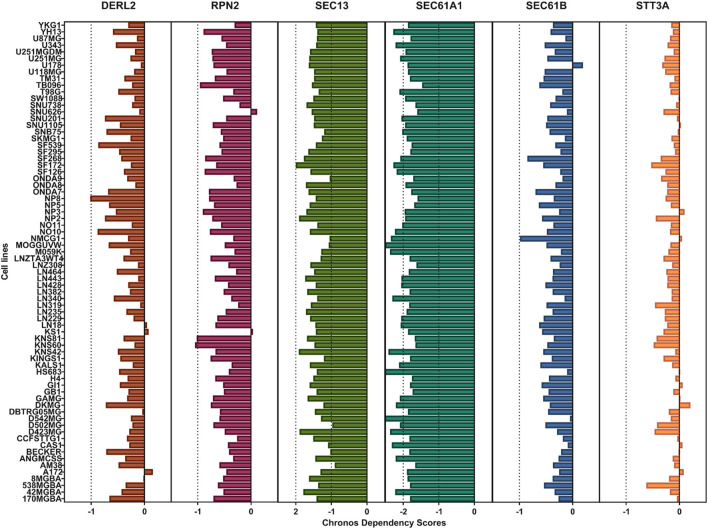
Glioma cells are preferentially dependent on genes comprising ESURATAG-GS for proliferation and survival. Bar-graphs showing CRISPR screen-based chronos dependency score of DERL2, RPN2, SEC13, SEC61A1, SEC61B and STT3A for 72 glioma cell lines.

Next, we aimed to identify key molecular hubs associated with ER stress and UPR-driven aging-related tumor aggressiveness in glioma. To this end, we combined the lists of genes associated with ER stress and UPR-related mechanisms in our pathway enrichment and functional annotation analysis (Figure 1C) and developed an interacting network among them using string database (Figure 3A). Six out of eight genes namely, DERL2, RPN2, SEC13, SEC61A1, SEC61B and STT3A were interacting, and were combined into ER stress and UPR-driven aging-related tumor aggressiveness in glioma (ESURATAG) gene signature. DERL2 represent a key component of UPR as it functions in ER-associated degradation (ERAD) pathway and retro-translocates misfolded proteins, thereby alleviating ER stress (Sugiyama et al., 2021). It has been implicated in cancer cell survival, tumor progression and chemotherapy resistance in cholangiocarcinoma via stabilizing BAG6 (Liu et al., 2024). RPN2, a subunit of the oligosaccharyltransferase complex, is essential for N-linked glycosylation, thereby playing a vital role in protein folding, stability and subsequent function (Han et al., 2023). Its overexpression has been reported to suppress radio-sensitivity by activating STAT3 signaling in gliomas (Li et al., 2020). SEC13 participates in COPII vesicle formation and nuclear pore assembly, maintaining protein trafficking and ER homeostasis (Anglès et al., 2024). Its dysregulation exacerbates ER stress and supports tumor proliferation (Yang et al., 2024). SEC61A1 and SEC61B form the core of the ER translocon complex responsible for co-translational protein translocation into the ER lumen (Itskanov and Park, 2023). Their overexpression is associated with increased secretory capacity and survival of cancer cells under ER stress conditions (Ji et al., 2024). Finally, STT3A, the catalytic subunit of the oligosaccharyltransferase, ensures proper N-glycosylation critical for protein folding (Mohanty et al., 2020). Its elevated expression has been shown to promote tumor progression in lung cancer via MAPK and PI3K signaling pathways (Cheng et al., 2022). Protein expression of genes in our ESURATAG-GS was also significantly upregulated in primary tumors compared to normal tissues in glioma patients (Figures 3B–F; protein expression data for DERL2 was not available). Notably, CRISPR screen-based data from 72 glioma cell lines also suggested that almost all of the cell lines were moderately-to-highly dependent on genes comprising ESURATAG-GS for proliferation and survival (Figure 4), further confirming the importance of ESURATAG-GS in glioma.

### 3.4 ESURATAG-GS confers disease progression, driving poor clinical outcomes in glioma patients

**FIGURE 5:**
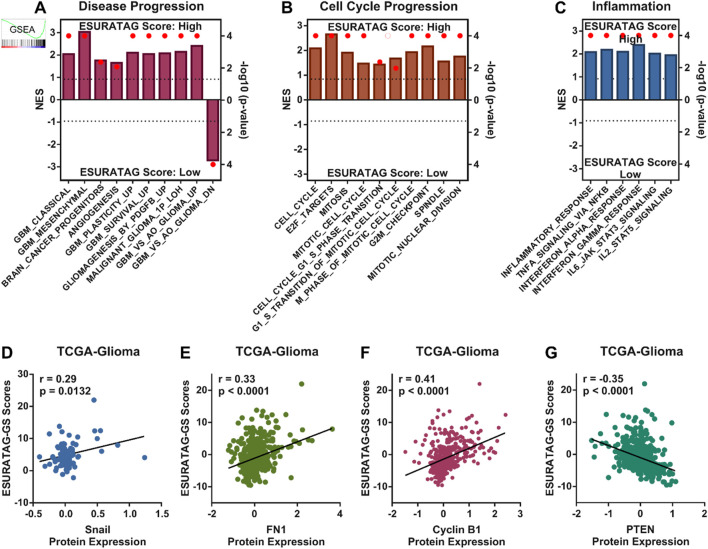
ESURATAG-GS confers disease progression in glioma patients. **(A–C)** Combined bar-dot-plots showing normalized enrichment scores (NES) (bars) and enrichment significance (dots) for GSEAs of disease progression-related **(A)**, cell cycle progression-related **(B)** and inflammation-related **(C)** gene signatures in glioma patients having low vs. high ESURATAG-GS scores in TCGA dataset. **(D–G)** Scatter-plots showing Pearson correlation of ESURATAG-GS scores with protein expression of Snail **(D)**, FN1 **(E)**, Cyclin B1 **(F)** and PTEN **(G)** in glioma patients from TCGA dataset.

**FIGURE 6:**
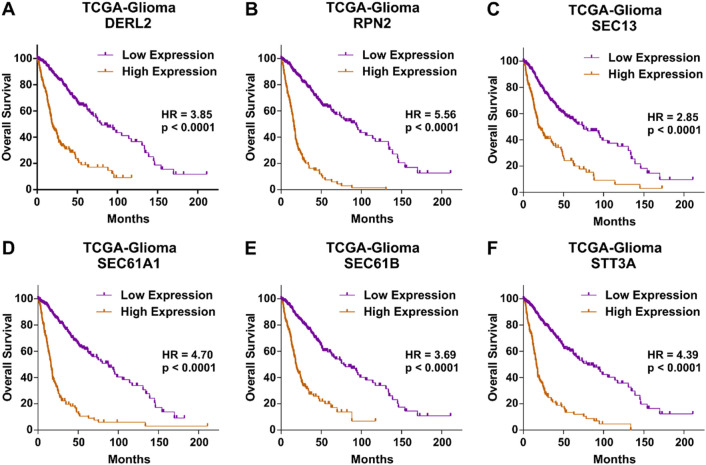
Genes comprising ESURATAG-GS are associated with poor survival in glioma patients. **(A–F)** Kaplan-Meier survival plots showing overall survival analysis based on low and high DERL2 **(A)**, RPN2 **(B)**, SEC13 **(C)**, SEC61A1 **(D)**, SEC61B **(E)** and STT3A **(F)** expression in glioma patients from TCGA dataset.

Next, we sought to understand whether our identified ESURATAG-GS is associated with different aspects of disease progression in gliomas. To this end, we performed GSEA and found that different gene sets associated with disease progression are enriched in glioma patients having high ESURATAG-GS scores compared to those having low scores (Figure 5A). Hyperactive cell cycle progression has been identified as key mechanisms behind glioma progression (Sun et al., 2024). In this line, we found that different gene sets associated with cell cycle progression are enriched in glioma patients having high ESURATAG-GS scores compared to those having low scores (Figure 5B). Inflammation is a key hallmark of cancer, in general, and of gliomas, in particular (Alorfi et al., 2024; Li et al., 2025). In this context, we found that different inflammation-related gene sets are enriched in glioma patients having high ESURATAG-GS scores compared to those having low scores (Figure 5C). In addition, ESURATAG-GS expression positively correlates with protein expression of tumor aggressiveness marker, Snail (Figure 5D) and FN1 (Figure 5E), confirming the role of ESURATAG-GS in tumor progression in gliomas. Moreover, ESURATAG-GS expression positively correlated with protein expression of cell cycle regulator, Cyclin B1 (Figure 5F) and negatively correlated with cell cycle inhibitor, PTEN (Figure 5E), confirming the role of ESURATAG-GS in cell cycle progression in gliomas. Notably, high expression of genes comprising ESURATAG-GS is associated with poor overall survival in gliomas (Figures 6A–F). These findings confirm that ESURATAG-GS confers disease progression and drives poor clinical outcomes in glioma patients.

### 3.5 MYC preferentially upregulates ESURATAG-GS in glioma patients

**FIGURE 7:**
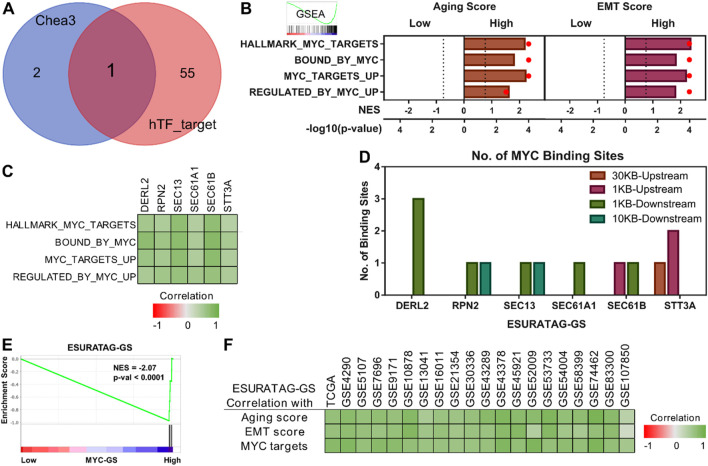
MYC preferentially upregulates ESURATAG-GS in glioma patients. **(A)** Venn diagram showing intersecting transcription factors (TFs) predicted to regulate ESURATAG-GS. Data from Chea3 and hTFtarget was utilized for analysis. **(B)** Combined bar-dot-plots showing normalized enrichment scores (NES) (bars) and enrichment significance (dots) of GSEAs of MYC targets-related gene signatures in glioma patients having low vs. high aging scores (left) and low vs. high EMT scores (right) in TCGA dataset. **(C)** Heatmap showing Pearson correlation of mRNA expression of genes comprising ESURATAG-GS with MYC targets-related gene signatures in TCGA dataset. **(D)** Bar-graph showing number of MYC binding sites in genes comprising ESURATAG-GS. **(E)** GSEA showing the enrichment of ESURATAG-GS in glioma patients having low vs. high MYC-GS scores in TCGA dataset. **(F)** Heatmap showing Pearson correlation of ESURATAG-GS scores with aging score, EMT score and MYC targets-related gene signature in TCGA and 19 different GEO datasets. EMT; epithelial-to-mesenchymal transition.

Next, we aimed to identify how ESURATAG-GS is regulated in gliomas. To this end, we checked the mutation status of genes comprising ESURATAG-GS, but found that these genes are not much mutated in gliomas (Supplementary Figure S2). Next, checked whether these genes are regulated through some common transcription factor (TF) in gliomas. To this end, we performed TF prediction analysis using online tools and found MYC as common TF predicted to regulate ESURATAG (Figure 7A). MYC has already been shown to regulated tumor aggressiveness in gliomas (Sun et al., 2024). We sought to check whether MYC-driven regulation concommitantly aligns with aging and tumor aggressiveness in gliomas. To this end, we performed GSEA and found that gene targets regulated by MYC are enriched in patients having high aging scores compared to those having low score, as well as in patients having high EMT scores compared to those having low scores (Figure 7B). Notably, gene sets comprising of genes potentially regulated by MYC are positively correlated with genes comprising ESURATAG-GS (Figure7C). Moreover, all the genes comprising ESURATAG-GS have at least one binding site for MYC (Figure 7D), suggesting their preferential regulation by MYC in gliomas. In line with this, ESURATAG-GS is enriched in glioma patients having high MYC-GS (comprising of list of genes potentially regulated by MYC) scores compared to those having low scores (Figure 7E). Finally, ESURATAG-GS exhibit high positive correlation with aging and EMT scores, and MYC-driven targets in 20 different glioma patient datasets (Figure 7F), confirming that MYC-driven ESURATAG-GS lies at the heart of aging-related tumor aggressiveness in glioma patients.

### 3.6 ESURATAG-GS is associated with disease onset and progression in glioma patients

**FIGURE 8:**
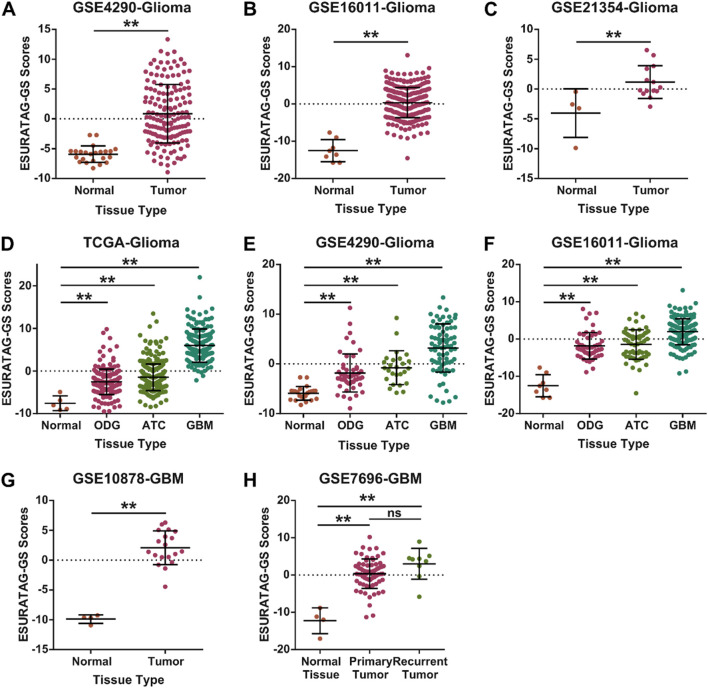
ESURATAG-GS is associated with disease onset and progression in glioma patients. **(A–C)** Dot-plots showing changes in ESURATAG-GS expression between normal and primary tumor tissues in glioma patients from GSE4290 **(A)**, GSE16011 **(B)** and GSE21354 **(C)** datasets. **(D–F)** Dot-plots showing changes in ESURATAG-GS expression in different subtypes of gliomas (ODG, ATC, GBM) as compared to normal tissues from TCGA **(D)**, GSE4290 **(E)** and GSE16011 **(F)** datasets. **(G)** Dot-plot showing changes in ESURATAG-GS expression between normal and primary tumor tissues in GBM patients from GSE10878 dataset. **(H)** Dot-plot showing changes in ESURATAG-GS expression among normal, primary tumor and recurrent tumor tissues in GBM patients from GSE7696 dataset. ATC; astrocytoma, GBM; glioblastoma, ODG; oligodendroglioma. ns: non-significant, *: p < 0.05, **: p < 0.01.

Next, we aimed to validate our findings in independent datasets. We found that ESURATAG-GS expression is upregulated in primary tumors compared to normal tissues in multiple GEO datasets including GSE4290 (Figure 8A), GSE16011 (Figure 8B) and GSE21354 (Figure 8C), which have gene expression data from gliomas vs. normal tissue samples. Gliomas are diverse group of tumors which are now classified into three major subtypes; oligodendroglioma (ODG) which are isocitrate-dehydrogenase (IDH)-Mutant (Mut) along with 1p/19q co-deletion, astrocytoma (ATC) which are IDH-Mut without 1p/19q co-deletion and GBM which are IDH wildtype (WT). Among these, ODG is the least aggressive subtype whereas GBM is the most aggressive one (Park et al., 2023). In this lines, we sought to explore the glioma subtype-specific clinical implications of ESURATAG-GS, and found that ESURATAG-GS expression is sequentially upregulated among glioma subtypes from ODG to GBM compared to normal tissues in TCGA dataset (Figure 8D), and in two independent GEO datasets including GSE4290 (Figure 8E) and GSE16011 (Figure 8F). In addition, ESURATAG-GS expression is also upregulated in primary GBM tumors compared to normal tissues in GSE10878 (Figure 8G), and in recurrent and primary GBM tumors compared to normal tissues in GSE7696 dataset (Figure 8H). These findings affirm that ESURATAG-GS is associated with disease onset and progression in glioma patients.

### 3.7 ESURATAG-GS is associated with aggressive disease state in glioma patients

**FIGURE 9:**
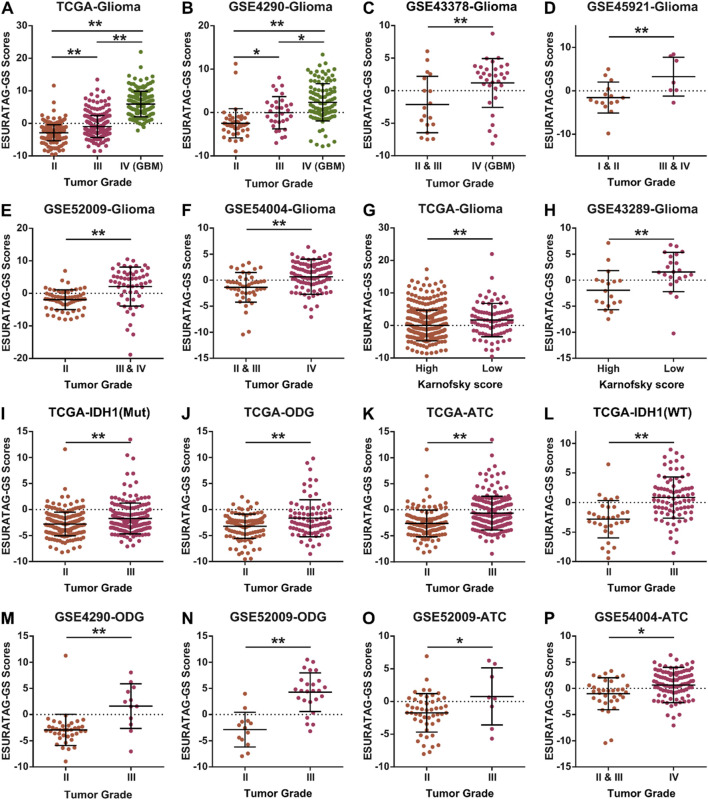
ESURATAG-GS is associated with aggressive disease state in glioma patients. **(A–F)** Dot-plots showing changes in ESURATAG-GS expression among different tumor grades in glioma patients from TCGA **(A)**, GSE4290 **(B)**, GSE43378 **(C)**, GSE45921 **(D)**, GSE52009 **(E)** and GSE54004 **(F)** datasets. **(E)** Dot-plots showing changes in ESURATAG-GS expression between tumors associated with high and low Karnofsky score in glioma patients from TCGA **(G)** and GSE43289 **(H)** datasets. **(I–L)** Dot-plots showing changes in ESURATAG-GS expression between different tumor grades in IDH1-Mut tumors **(I)**, ODGs **(J)**, ATCs **(K)** and IDH1-WT tumors **(L)** in TCGA dataset. **(M–P)** Dot-plots showing changes in ESURATAG-GS expression between different tumor grades in ODGs from GSE4290 (Figure 9M) and GSE52009 (Figure 9N) datasets, and in ATCs from GSE52009 (Figure 9O) and GSE54004 (Figure 9P). ATC; astrocytoma, ODG; oligodendroglioma. *: p < 0.05, **: p < 0.01.

Next, we aimed to confirm whether ESURATAG-GS expression is associated with clinical aspects of disease in glioma patients. In this line, we found that high ESURATAG-GS expression is associated with higher tumor grade in TCGA-Glioma dataset (Figure 9A) and multiple GEO datasets including GSE4290 (Figure 9B), GSE43378 (Figure 9C), GSE45921 (Figure 9D), GSE52009 (Figure 9E) and GSE54004 (Figure 9F). Karnofsky score is a scoring plan for the performance of cancer patients in daily life, better the performance, higher the score (Péus et al., 2013). We found that ESURATAG-GS expression is high in patients with low Karnofsky score in TCGA-Glioma (Figure 9G) and GSE4389 (Figure 9H) datasets, suggesting that ESURATAG-GS expression is associated with poor performance in glioma patients. Regarding glioma subtype-specific clinical implications, we found that ESURATAG-GS expression is associated with higher tumor grade in IDH1-Mut tumors (Figure 9I), ODGs (Figure 9J), ATCs (Figure 9K) and IDH1-WT tumors (Figure 9L) in TCGA dataset. In addition, ESURATAG-GS expression is also found to be associated with higher tumor grade in ODGs in two independent GEO datasets, GSE4290 (Figure 9M) and GSE52009 (Figure 9N). Similarly, ESURATAG-GS expression is also found to be associated with higher tumor grade in ATCs in two independent GEO datasets, GSE52009 (Figure 9O) and GSE54004 (Figure 9P). Better therapeutic response towards alkylating agents has been attributed to O^6^-methylguanine-DNA methyltransferase (MGMT) promoter hypermethylation in glioma patients, particularly those with GBM subtype (Bobola et al., 2015). In this line, we found that ESURATAG-GS score is negatively correlated with MGMT gene methylation score in glioma patients from TCGA dataset (Supplementary Figure S3). These findings affirm that ESURATAG-GS is associated with aggressive disease state in glioma patients.

### 3.8 ESURATAG-GS is associated with poor survival in glioma patients

**FIGURE 10 F10:**
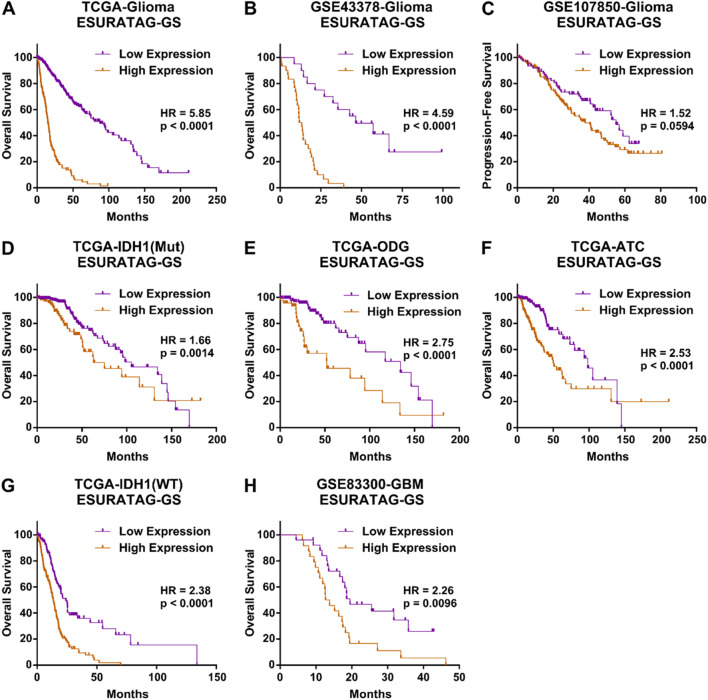
ESURATAG-GS is associated with poor survival in glioma patients. **(A,B)** Kaplan-Meier survival plots showing overall survival analysis based on low and high ESURATAG-GS expression in glioma patients from TCGA **(A)** and GSE43378 **(B)** datasets. **(C)** Kaplan-Meier survival plot showing progression-free survival analysis based on low and high ESURATAG-GS expression in glioma patients from GSE107850 dataset. **(D–G)** Kaplan-Meier survival plots showing overall survival analysis based on low and high ESURATAG-GS expression in IDH1-Mut tumors **(D)**, ODGs **(E)**, ATCs **(F)** and IDH1-WT tumors **(G)** from TCGA dataset. **(H)** Kaplan-Meier survival plot showing overall survival analysis based on low and high ESURATAG-GS expression in GBM patients from GSE83300 dataset. ATC; astrocytoma, GBM; glioblastoma, ODG; oligodendroglioma.

Lastly, we aimed to validate whether ESURATAG-GS expression is associated with clinical outcome in glioma patients. To this end, we performed survival analysis of ESURATAG-GS in different patients datasets and found that, in addition to TCGA-Glioma dataset (Figure 10A), high expression of ESURATAG-GS is also associated with poor overall survival in glioma patients in GSE43378 (Figure 10B). In addition, high expression of ESURATAG-GS is also associated with poor progression-free survival in glioma patients from GSE107850 (Figure 10D). Regarding subtype-specific analysis, we found that high expression of ESURATAG-GS is associated with poor overall survival in IDH1-Mut tumors (Figure 10D), ODGs (Figure 10E), ATCs (Figure 10F) and IDH1-WT tumors (Figure 10G) in TCGA dataset. Moreover, high expression of ESURATAG-GS is also associated with poor overall survival in GBM patients in GSE83300 (Figure 10H). Overall, these findings affirm that ESURATAG-GS is associated with poor survival in glioma patients.

## 4 Discussion

**FIGURE 11:**
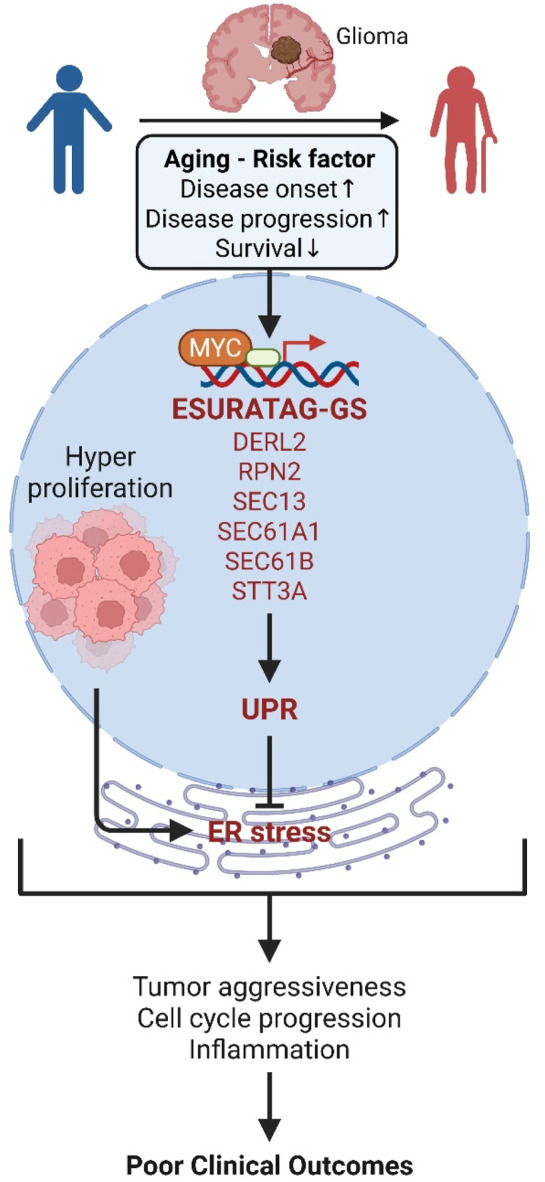
Schematic summary. Aging is a significant risk factor for glioma incidence and progression, and is associated poor survival in glioma patients. As the tumors grow, hyper-proliferation of cancer cells induce ER stress. However, aging-related MYC drives the expression of ESURATAG-GS, including DERL2, RPN2, SEC13, SEC61A1, SEC61B and STT3A, which orchestrates UPR to counteract ER stress. This contributes to tumor aggressiveness, cell cycle progression and inflammation, resulting in aging-related poor clinical outcomes in glioma patients. UPR; unfolded-protein response.

Gliomas remain a formidable clinical challenge due to their aggressive nature, heterogeneity, and limited therapeutic options, especially in elderly patients where aging-associated molecular alterations contribute significantly to tumor progression (Sipos and Raposa, 2025; Zhang et al., 2025a). Here, we identified a novel ER stress and UPR-driven gene signature, ESURATAG, composed of six genes (DERL2, RPN2, SEC13, SEC61A1, SEC61B, and STT3A) that are significantly upregulated in gliomas from older patients and strongly linked to tumor aggressiveness. Differential expression analyses across TCGA showed ESURATAG expression increases with aging, tumor grade, EMT scores, and disease progression, including recurrent tumors (Figure 1). GSEA confirmed enrichment of ER stress and UPR pathways in aging and tumor aggressiveness groups (Figure 2). Protein expression data and CRISPR dependency screens further validated the importance of ESURATAG genes for tumor proliferation and survival (Figures 3,4 [Fig F4]). Clinically, high ESURATAG expression associates with disease onset, advanced and aggressive disease state, poor Karnofsky scores, and worse overall and progression-free survival (Figures 5,6,8–10). MYC was identified as a key transcriptional regulator of ESURATAG, with strong correlations to aging and EMT signatures, indicating MYC-driven ER stress adaptation fuels aging-related glioma progression (Figure 7). These findings establish ESURATAG as a critical biomarker and potential therapeutic target in elderly glioma patients (Figure 11).

The ER stress response and UPR are vital cellular processes that maintain protein homeostasis, especially under conditions of proteotoxic stress common in rapidly proliferating tumor cells (Spencer and Finnie, 2020). Our findings of aging-related upregulation of ESURATAG-GS containing DERL2, RPN2, SEC13, SEC61A1, SEC61B and STT3A (Figures 1,3 [Fig F3]) highlight a robust adaptive mechanism to sustained proteostasis challenges. As discussed earlier, each of these genes plays a specialized role in ER protein quality control, degradation of misfolded proteins, glycosylation, and protein translocation, collectively enhancing glioma cells’ capacity to survive and proliferate despite adverse microenvironmental conditions (Li et al., 2020; Mohanty et al., 2020; Sugiyama et al., 2021; Cheng et al., 2022; Han et al., 2023; Itskanov and Park, 2023; Anglès et al., 2024; Ji et al., 2024; Liu et al., 2024; Yang et al., 2024). Collectively, these processes highlight ER stress and UPR as indispensable adaptive responses in aging gliomas, consistent with findings that chronic ER stress promotes tumor progression and therapy resistance (Wang and Mi, 2023). In addition, proteostasis has emerged as a key molecular mechanism in glioma biology, with heparanase (HPSE) shown to promote autophagy and support tumor progression by regulating intracellular stress responses (Kundu et al., 2016). This mechanistic insight aligns with our findings, where high ESURATAG expression, enriched for proteostasis and UPR-related genes, correlates with increased tumor aggressiveness. Notably, other than RPN2, which has been shown to promote radioresistance via upregulating STAT3 signaling in gliomas (Li et al., 2020), none of the genes in ESURATAG-GS have been studied in the context of glioma, highlighting the novelty of our findings.

Our data identify MYC as a pivotal transcriptional regulator of the ESURATAG gene set (Figure 7), aligning with previous research establishing MYC’s role in driving tumor onset and progression in gliomas (Annibali et al., 2014; Sun et al., 2024). MYC’s ability to transcriptionally activate ER stress response genes positions it as a crucial node integrating proliferative signaling with proteostasis mechanisms, particularly in the context of aging-associated glioma aggressiveness (Figure 7). This is consistent with reports demonstrating MYC’s involvement in UPR regulation and its contribution to cancer development and progression (Zhang et al., 2020). MYC has been shown to regulate cell cycle progression-driven tumor aggressiveness in gliomas (Sun et al., 2024), aligning with our findings that cell cycle progression-related gene signatures are enriched in patients having high ESURATAG-GS scores (Figure 5B). Our findings complement the notion of MYC’s involvement in cell cycle progression in gliomas and expand the scope of MYC-driven oncogenic programs to include ER stress and proteostasis pathways. Together, these studies delineate a comprehensive picture wherein MYC orchestrates multiple adaptive processes including metabolic reprogramming, DNA replication, and ER stress responses that converge to promote tumor growth, progression, and treatment resistance, particularly in aging gliomas. The strong correlation between MYC targets, ESURATAG expression, EMT markers and aging signature (Figure 7E) further suggests that MYC-driven ER stress adaptation not only supports proliferation and progression, but may also promote tumor aggressiveness in an-aging dependent manner as MYC is being actively recognized as a crucial factor in driving aging-related cancer onset and progression (Hofmann et al., 2015;Wang et al., 2023).

We demonstrated that high ESURATAG expression correlates with advanced tumor grade, poor Karnofsky performance status, and worse overall and progression-free survival across multiple independent glioma cohorts (Figures 8–10). These findings position ESURATAG as a robust molecular biomarker reflective of both intrinsic tumor biology and the systemic impact of aging. In addition to its association with aging and tumor progression, our findings demonstrate that ESURATAG-GS exhibits distinct expression patterns across the three major glioma subtypes observed in adults (as defined by WHO 2021 classification of the tumors of central nervous system; ODG, ATC and GBM (Louis et al., 2021), with a progressive increase from ODG to GBM (Figure 8), consistent with their known clinical aggressiveness (Park et al., 2023). This gradient of ESURATAG expression suggests a subtype-specific role of ER stress adaptation in driving malignancy. Importantly, high ESURATAG-GS expression correlated with aggressive disease state and poor survival not only in GBM, but also within ODG and ATC subtypes (Figures 9,10 [Fig F10]) indicating that this gene set may capture aggressiveness traits even in traditionally less aggressive gliomas. Given the limited prognostic power of conventional histopathological grading alone, incorporating molecular signatures such as ESURATAG could enhance subtype-specific risk stratification and guide personalized therapeutic approaches, particularly in the elderly glioma population (Ghosh and Patel, 2025). The observed functional dependency of glioma cell lines on ESURATAG genes in CRISPR screens (Figure 4) underscores their potential as therapeutic targets. Interventions disrupting ER stress adaptation pathways have shown promise in preclinical cancer models, and targeting ESURATAG components may sensitize gliomas to existing therapies or novel agents designed to induce lethal ER stress (Xipell et al., 2016). Moreover, the inverse correlation between ESURATAG-GS and MGMT promoter methylation in gliomas (Supplementary Figure S3), a key determinant of therapeutic response (Bobola et al., 2015), suggests that tumors with high ER stress burden may be more resistant to standard alkylating chemotherapy, reinforcing the therapeutic relevance of this pathway.

While our integrative bioinformatics approach reveals novel insights into aging-related glioma biology, several limitations warrant consideration. First, the observational nature of transcriptomic and proteomic associations presented in this study necessitates functional validation through *in vitro* and *in vivo* experiments to confirm causality and mechanistic details of ESURATAG genes in glioma progression. Second, the heterogeneity of patient cohorts and potential confounding variables like molecular sub-classification of GBMs based on markers, such as, TERT promoter mutations and EGFR gene amplification, may influence gene expression patterns and should be accounted for in future studies (Louis et al., 2021). Third, the therapeutic targeting of ER stress and UPR components poses challenges due to the essential physiological roles of these pathways in normal cells; thus, strategies must aim for tumor-selective modulation (Yuan et al., 2024). Future work should focus on experimental perturbation of ESURATAG genes in glioma models to evaluate their contribution to tumor growth, invasiveness, and treatment response, particularly in aged microenvironments. Additionally, integrating single-cell transcriptomics could unravel cell-type-specific ER stress adaptations, offering refined targets for therapeutic interventions (Longo and Guo, 2021). Ultimately, assessing ER stress modulators in molecularly defined glioma subgroups stratified by aging and ESURATAG expression may provide novel avenues for improving outcomes in this challenging patient population.

## 5 Conclusion

In conclusion, we identify a novel ER stress and UPR-driven gene signature, ESURATAG that plays a critical role in aging-related tumor aggressiveness in gliomas. This signature, tightly regulated by MYC, enables tumor cells to adapt to proteotoxic stress and supports their proliferation and survival. The strong correlation of ESURATAG expression with disease onset, tumor grade, patient performance, and poor survival underscores its potential as both a prognostic biomarker and a therapeutic target. Our findings highlight the importance of targeting ER stress adaptation pathways to improve treatment outcomes, especially in elderly glioma patients.

## Data Availability

The datasets presented in this study can be found in online repositories. The names of the repository/repositories and accession number(s) can be found in the article/Supplementary Material.
