# Supplementary material for: ER stress-driven unfolded protein response fuels aging-related tumor aggressiveness in gliomas
Source: Front Mol Biosci. 2025 Jul 11;12:1640038. doi: 10.3389/fmolb.2025.1640038 (PMC12289514; doi:10.3389/fmolb.2025.1640038)
Supplement: Supplementary file 1 [file DataSheet1.pdf]

ER stress-driven unfolded protein response fuels aging-related tumor aggressiveness in gliomas

Supplementary Figures

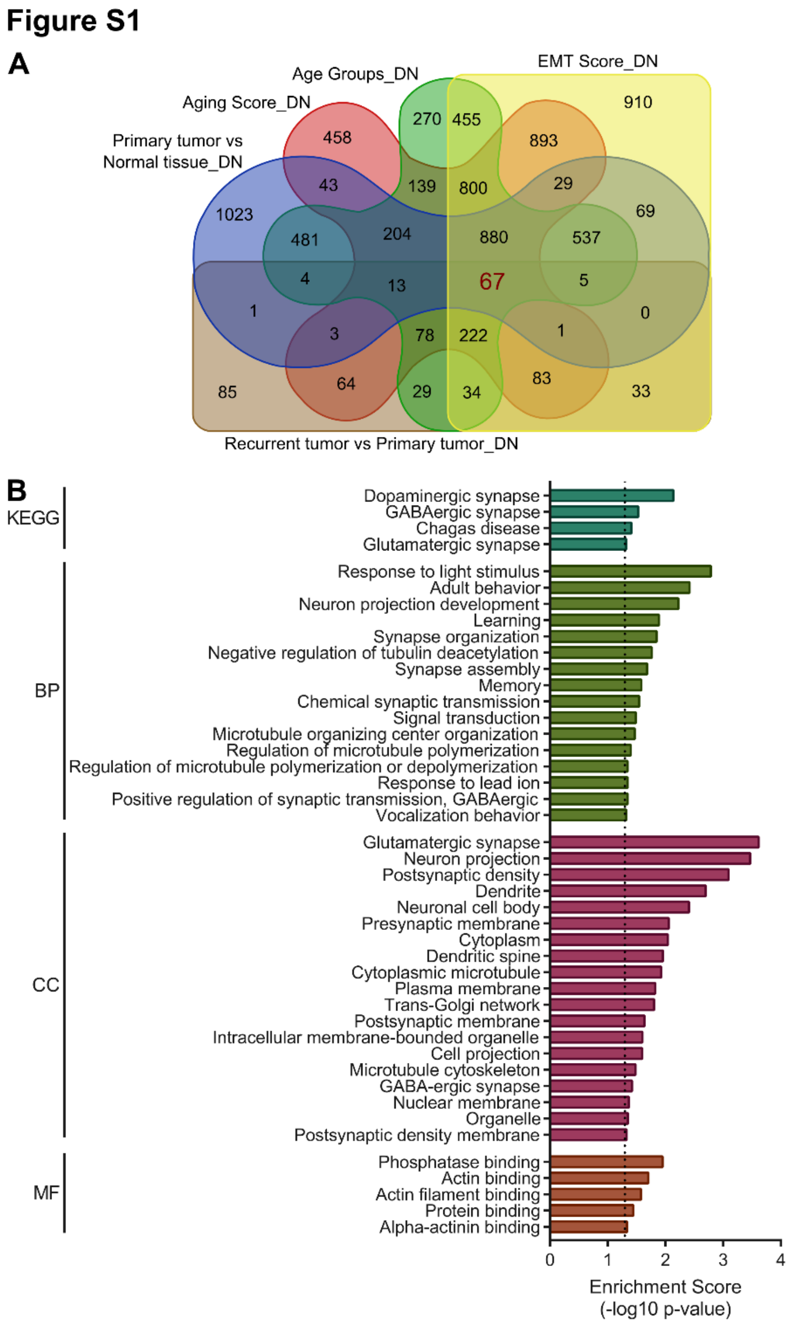

Supplementary Figure S1: Aging-related tumor aggressiveness-targeted expression analysis identifies brain function-related mechanisms as tumor suppressor pathways in glioma patients. (A) Venn diagram showing number of aging-related tumor aggressiveness-associated tumor suppressor hubs through pipeline in Figure 1A. (B) Bar-graph showing enrichment score of KEGG pathways, biological processes, cellular compartments and molecular functions in which tumor suppressor hubs, identified in (A), are enriched.

Figure S2

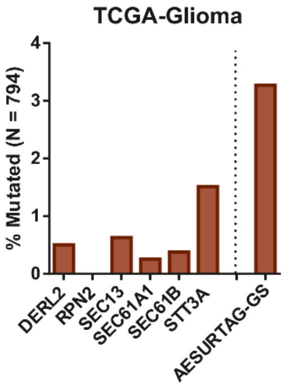

Supplementary Figure S2. Genes comprising ESURATAG-GS are not mutated in glioma patients. Bar-graph showing number of glioma patients from TCGA dataset having mutations in genes comprising ESURATAG-GS.

Figure S3

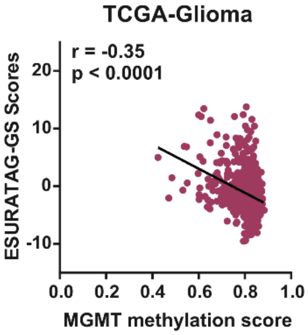

Supplementary Figure S3. ESURATAG-GS is inversely correlated with MGMT methylation in gliomas. Scatter-plot showing Pearson correlation of ESURATAG-GS scores with MGMT methylation scores in glioma patients from TCGA dataset.
